# Hypothalamic Neuropeptide Brain Protection: Focus on Oxytocin

**DOI:** 10.3390/jcm9051534

**Published:** 2020-05-19

**Authors:** Maria Antonietta Panaro, Tarek Benameur, Chiara Porro

**Affiliations:** 1 Department of Biosciences, Biotechnologies and Biopharmaceutics, University of Bari, 70125 Bari, Italy; mariaantonietta.panaro@uniba.it; 2 Department of Biomedical Sciences, College of Medicine, King Faisal University, 31982 Al-Ahsa, Saudi Arabia; tbenameur@kfu.edu.sa; 3 Department of Clinical and Experimental Medicine, University of Foggia, 71121 Foggia, Italy

**Keywords:** oxytocin, hypothalamic neuropeptide, microglia, neuroprotection

## Abstract

Oxytocin (OXT) is hypothalamic neuropeptide synthetized in the brain by magnocellular and parvo cellular neurons of the paraventricular (PVN), supraoptic (SON) and accessory nuclei (AN) of the hypothalamus. OXT acts in the central and peripheral nervous systems via G-protein-coupled receptors. The classical physiological functions of OXT are uterine contractions, the milk ejection reflex during lactation, penile erection and sexual arousal, but recent studies have demonstrated that OXT may have anti-inflammatory and anti-oxidant properties and regulate immune and anti-inflammatory responses. In the pathogenesis of various neurodegenerative diseases, microglia are present in an active form and release high levels of pro-inflammatory cytokines and chemokines that are implicated in the process of neural injury. A promising treatment for neurodegenerative diseases involves new therapeutic approaches targeting activated microglia. Recent studies have reported that OXT exerts neuroprotective effects through the inhibition of production of pro-inflammatory mediators, and in the development of correct neural circuitry. The focus of this review is to attribute a new important role of OXT in neuroprotection through the microglia–OXT interaction of immature and adult brains. In addition, we analyzed the strategies that could enhance the delivery of OXT in the brain and amplify its positive effects.

## 1. Introduction

The relationships among neurons, microglia and glial cells in the central nervous system (CNS) are very dynamic and responsive to different stimuli. In response to injury, infection or disease, the resident cells of the CNS produce cytokines, adhesion molecules, chemokines, free radicals and prostaglandins; these inflammatory mediators stimulate the enrollment of additional immune cells and enhance the activity of astrocytes and microglia. Hormones act as chemical messengers and play a key role in the maintenance of homeostasis in the body, acting within the brain and between the brain and the body. Oxytocin (OXT) is a nonapeptide produced in the paraventricular (PVN), supraoptic (SON) and accessory nuclei (AN) of the hypothalamus in the brain. OXT is conducted by axonal transport to the posterior pituitary, where it is accumulated in neurosecretory granules before its release into the bloodstream. OXT, released in response to different physiological stimuli, plays an important role in reaction to stress and in reducing proinflammatory responses in various contexts such as neurodegenerative disorders [1]. Neurodegenerative diseases—including Alzheimer’s Disease (AD), Parkinson’s Disease (PD) and Amyotrophic Lateral Sclerosis (ALS), as well as Huntington’s disease—are main causes of death, and entail human suffering, social distress and economic burden.

Neurodegenerative diseases are pathological conditions associated with aging, but neurodegeneration often hides in a subclinical form for a long time, and neuronal death occurs progressively over a lifetime, before the first clinical signs can be observed. Different clinical profiles represented by abnormal protein deposition, dysfunctional cellular transport, mitochondrial deficits, glutamate excitotoxicity, iron accumulation and inflammation are characterized by neurodegeneration and neuronal loss. These features are observed in different neurodegenerative disorders and share a common cascade of molecular signaling as a result of the activation of microglia by signals of damage.

Many neurodegenerative diseases are associated with specific pathways, and share common neuroinflammatory pathways [2] and oxidative stress mechanisms [3]. These inflammatory pathways play a pivotal molecular basis in the pathogenesis of the neurodegenerative diseases; yet the exact cellular mechanisms for neurodegeneration are complex and not fully understood.

At the moment, it is important to stress that there are no effective therapies available for treating neurodegenerative diseases other than the amelioration of symptoms [4,5]. Indeed, neuroprotection remains one of the main strategies that can delay or prevent the onset of these diseases.

OXT plays an important role in endocrine and paracrine activities such as the development of social recognition, sexual and maternal behaviors, neuron modulation, aggression, cognition and broad-mindedness [6]. Recent investigations on animal models have provided evidence that OXT has anti-inflammatory and anti-oxidant properties and modulates immune and anti-inflammatory responses [7,8].

The purpose of the current review article is to describe the protective role of OXT in the brain, with a particular focus on its direct action on microglial cells and function of the brain. A particular focus is attributed to the new important role of OXT in neuroprotection; OXT’s contributions to various physiological functions and psychological disorders are reviewed and highlighted. Furthermore, the potential use of OXT as a therapeutic agent is discussed and the strategies to enhance its delivery in the brain are analyzed.

## 2. Oxytocin-System: Oxytocin Synthesis, Release and its Receptor

Oxytocin is expressed firstly as an inactive precursor by the *OXT* gene located on chromosome 20. After undergoing a series of post-translational hydrolysis, this inactive precursor is converted to an active OXT [9]. OXT is synthesized along with its carrier protein neurophysin I. OXT is put into vesicles and axonally carried into the nerve endings in the neurohypophysis, where it is either stored or released into the bloodstream [10,11].

OXT is also synthesized in various peripheral tissues such as placenta, uterus, corpus luteum, amnion, testis, thymus, pancreas and kidney [12].

OXT binds to the oxytocin receptor (OXTR). The OXTR is a 389-amino acid belonging to the G-protein-coupled transmembrane receptor superfamily [13]. The human *OXTR* gene is located on chromosome 3 at locus 3p25.

The OXTR is bound by OXT, a neuropeptide composed of nine amino acids (Cys-Tyr-Ile-Gln-Asn-Cys-Pro-Leu-Gly). The secondary structure of OTX is formed by a ring, with a disulphide connection between the first and sixth cysteine, and has a very short tail comprising three amino acids. Studies conducted in an in vitro analyzing homology model, a test of mutagenesis and pharmacological aspects [14,15,16,17,18] have shown that while the tail interacts with the OXTR regions exhibited in an extracellular space, the cyclic part reaches the deep part of a receptor’s transmembrane core, where it has an interaction with residues located in TMH5 and TMH6.

The site of oxytocin receptor mRNA in the brain offers a possibility to see the distribution of oxytocin binding [19], providing an accurate map of the anatomical geography of the oxytocin system in the brain. Seminal animal researches using histochemical and immunohistochemical techniques have shown high concentrations of OXTR mRNA in the hypothalamus, ventral pallidum, amygdala, olfactory bulb and the dorsal vagal nucleus in rodents [20,21]. OXTR mRNA sites in rodent brains re well-known [22], but their anatomical distribution across the human brain is poorly understood.

A growing body of evidence suggests that changes in the epigenetic regulation of the oxytocin receptor gene gives the oxytocin system flexibility in response to various events, particularly in childhood. Indeed, a large number of recent studies have shown a single-nucleotide polymorphism association in oxytocin receptor genes and the social behavior and pathogenesis of psychiatric disorders [23].

Expression and binding of the OXTR in rat and mouse brains using radiolabeled ligand have been described previously [24]. Recently, a binding approach showed that commercially available radioligands can be used in combination with selective competitors to reveal specific distributions of both OXTR in postmortem human brain tissue. However, despite recent efforts, there are currently no in vivo neuroimaging agents for OXTR for PET imaging (Positron Emission Tomography), a technique that would be invaluable in research using humans and nonhuman primates) [25].

The activation of OXTR is associated with the activation of a number of various intracellular cascades of events that facilitate the OXT pathway [12]. OXTR is expressed within the peripheral organs and centrally in the brain [26]. Quintana et al. very recently identified the anatomical distribution of the oxytocin pathway of mRNA expression in the human brain in order to identify its functional importance and supposed gene interactions. They found that the oxytocin signaling system acts synergistically with dopaminergic and muscarinic acetylcholine signaling systems by exerting its complex effects on comprehension [27].

The OXT pathway is a multifaceted system associated with various biological functions. An increasing number of studies have reported the importance of the oxytocin system in the human development [28,29], modulation of pain processing [30] and feeding behaviors [31]. OXT has an important role in bird-related phenomena, and it is normally used clinically for the induction and increase of labor [20]. OXT is implicated in the contraction of smooth muscles during parturition and lactation. OXT is also involved in tolerance, cognition, adaptation and sexual and maternal behavior, as well as in the regulation of cardiovascular functions [32].

In addition, OXT was found in many areas of the brain and functions as a neurotransmitter that modulates a variety of social behaviors such as social cognition, nurturing and social bonding [33,34,35,36,37]. Further, OXT has been shown to have both antidiuretic and vasodilatory effects leading to an increased cerebral, coronary and renal blood flow [38,39,40].

## 3. The Role of Microglia in Neuroinflammation

Microglia, the resident macrophages of the brain and spinal cord, represent the key defense of the innate immune system. Microglia belong to the glial system of non-neuronal cells, which support and protects neuronal functions, including synaptic pruning and the phagocytosis of cellular debris [41,42]. Microglia derive from progenitors that begin in the yolk sac [43,44] and later proliferate and modify the developing CNS [45]. Microglia typically exist in a resting state, recognizable by a small cell body with fine, ramified processes that present a low expression of surface antigens and continuously monitor the brain parenchyma to preserve the proper homeostasis of the nervous tissue [46]. Following brain injury or infection, activated microglia—undergoing dramatic phenotypic changes toward an amoeboid morphology—retract their branches and exhibit a different antigenic profile. Activated microglia migrate toward toxic stimuli and promote an inflammatory response capable of producing a number of mediators and trophic factors that are essential to assuring tissue repair and maintenance of normal brain networking [46]. Considering that the activation of microglia is not a single event and that different “states of activation” have been attributed to these multifunctional cells, the M1/M2 paradigm has been introduced to simplify two opposite phenotypes described as proinflammatory M1 (classical activation) and immunosuppressive M2 (alternative activation; also known as tumor-supportive phenotype) [47,48]. M1 microglia polarization (also referred as neurotoxic phenotype) is correlated with the production and secretion of a number of proinflammatory cytokines, including tumor necrosis factor-alpha (TNF-α), IL-1β, IL-6 and IL-12, which in general react defend tissue and enhance the destruction of pathogens [47,48]. However, the overactivation or dysregulation of M1 microglia phenotype may amplify neuronal damage elicited by pathological stimuli and toxins, determining more widespread damage to the neighboring neurons [49].

Unlike M1 phenotype, alternative activation (M2-like) of microglia triggers upregulation of anti-inflammatory genes, including mannose receptor (CD206), arginase-1 (source of proline and polyamines), FIZZ and YM1. Alternative activation of microglia contributes to tissue repair and extracellular matrix reconstitution, thus promoting wound healing and tissue repair [50].

Alternative activation has been subdivided into two subcategories: M2a and M2b. M2a phenotype is achieved upon treatment with IL-4 or IL-13, exhibits an increased phagocytic activity and produces trophic polyamines, insulin-like growth factor-1 and anti-inflammatory cytokines such as IL-10. M2a is also able to express G-CSF, CD209 and GM-CSF [51,52,53]. This microglia population is able to remove the debris of cells and help tissue regeneration. M2b microglia phenotype is induced by the ligation of immunoglobulin Fc-gamma-receptors, resulting in increased IL-12 and IL-10 release and increasing CD64, CD32 and HLA-DR expression [54].

Finally, an acquired deactivation of polarized M2c was reported, induced by the anti-inflammatory cytokine IL-10 or glucocorticoids, which are an expanded expression of transforming growth factor (TGF) [55]. Since the polarization of microglia toward the M2 phenotype is able to solve inflammation and degeneration, this phenotype is described as neuroprotective even if it is also true that M2 phenotype has an absolutely divergent role in cases of neoplastic development playing a pro-tumoral role in the CNS [56]. Taken together, it is clear that these cells exhibit a pivotal role during inflammatory processes associated with neurodegenerative diseases.

Traditionally, inflammation represents a physiological immune response against different factors such as infection, trauma and disease to assure the integrity of the host [57]. In such cases, immune cells are recruited to the area of the insult, thus initiating a number of activities represented by the recruitment of other immune cells and the release of pro-inflammatory mediators, including transcription factors (e.g., NF-κB), cytokines (e.g., IFN-γ, IL-18, TNF-α, IL-1β, IL-6), chemokines (e.g., CXCL8, CCL2, CCL3,), complement components (e.g., C1q, C5), enzymes (e.g., iNOS, COX-2, LOX), lipids (e.g., PGE2) and coagulation factors (e.g., platelet activating factor) [52]. When the insult is neutralized, immune responses shift towards a phenotype triggering the resolution via the upregulation of anti-inflammatory mediators, with the purpose of promoting clearance and repair of the injured tissue. These mediators comprise lipoxins (e.g., LXA4, RvE1) and anti-inflammatory cytokines (e.g., IL-10, IL-37, TGF-β). When resolution is not achieved, the inflammatory responses persist longer than necessary, are often described as long-lasting and self-perpetuating and can become chronic. In this case chronic inflammation, rather than playing a protective role and promoting the elimination of pathogens, leads to tissue damage [58,59].

Besides the involvement of endothelial cells, astrocytes and peripherally derived immune cells in mediating these responses, microglial cells play a pivotal role in propagating neuroinflammatory signals due to their crucial role in the immune surveillance of the CNS [60,61].

In this regard, microglia also activate astrocytes that in turn can modulate the activation and recruitment of other immunocompetent cells to the injury site, thus contributing to exacerbating the inflammatory picture or, alternatively, to promoting the resolution of the neuroinflammation process [62]. The involvement of astrocytes in neuroinflammation has been clearly demonstrated by a considerable number of in vitro and in vivo studies. Indeed, increased expression of glial fibrillary acidic protein (GFAP), which is commonly considered as a hallmark of neuroinflammation in various neurodegenerative conditions, has been found [63]. However, by representing danger-signal sensors due to the expression of TLR4, microglia play a critical role in the establishment and maintenance of inflammatory responses during neurodegenerative diseases [64].

Although neuroinflammation may not typically represent itself an initiating factor in neurodegenerative disease, evidence suggests that increased inflammatory responses involving microglia activation contribute to disease development [65,66,67]. The connection present between neuroinflammation, microglial activation and neuronal death has been also studied in many neurodegenerative diseases, including AD and PD [68,69]. In addition, marked microgliosis has been observed in post-mortem patients affected by neurodegenerative disease, including Huntington disease, AD and PD [70,71].

A fundamental and still unanswered question is whether the inhibition of the microglia responses can represent a useful and effective tool to stop or slow down the course of neurodegenerative diseases.

For example, some studies have reported that negative feedback mechanisms may attenuate microglia responses against proinflammatory inducers, among these the Suppressors of Cytokine Signaling Proteins (SOCS), as well as during the production of soluble or cell-surface mediators with anti-inflammatory activities (such as TGFα, IL-10, ligands for TAM receptors). SOCS have been reported as possible [72,73].

In this context, recent studies have put attention on the fundamental role of the PI3K/Akt cascade in the microglial activation pathway and during neuroinflammation, and modulation of the PI3K/Akt axis has been proposed as a promising therapeutic approach for many neuropathological disorders [74].

Prior research suggests that microglia can be modulated in order to contain inflammatory processes driving immune responses in the brain in order to procure and promote beneficial effects [75,76].

Therefore, manipulation of the balance between protective and degenerative neuroinflammation is gaining a therapeutic potential against several neurodegenerative diseases. In particular, a number of recent studies have demonstrated that different approaches can selectively drive the polarization of microglia in order to regulate the synthesis of mediators toward a neuroprotective action to prevent neurodegeneration [77,78]. Substantially, additional studies must be conducted to better explore the capacities of microglia to resolve neuroinflammation.

## 4. Effect of Oxytocin on Microglia

Brain inflammatory responses are orchestrated by the crosstalk between astrocytes and microglia [79]. Although microglia have long been considered to be proinflammatory cells promoting neuronal toxicity and death, recent studies have demonstrated that these cells play a pivotal role in a number of physiological conditions.

Microglia express various sets of receptors including adrenergic α1, purinergic P2Y, P2X, thrombin, platelet-activating factor, endothelin, cytokine and chemokine [80,81]. Other evidence from the literature has demonstrated that microglia express oxytocin receptor that contributes to the regulation and control of microglia functions to varying degrees. [82,83,84].

Most of the current evidence supports OXT potentially having an important role in the CNS. Indeed, one finding that is strongly supported is that OXT concentration can be up to 1000× higher in the brain than in the peripheral blood [85]. Interestingly, recent studies have demonstrated that OXT has gained attention as a potent modulator of both synaptic plasticity and adult-born neurogenesis [86,87]. In this regard, it has been reported that OXT controls adult hippocampal neurogenesis through an indirect non-cell autonomous mechanism by OXTR expressed in CA3 pyramidal neurons [88]. Recent findings have reported the important role of the oxytocin system on the early postnatal lives of mammals, which is useful for understanding the pathological processes of neurodevelopmental diseases and their compensations by early treatment [89].

OXT synthesis and release is restricted to PVN, SON and AN, but oxytocin positive cells have also been found in the subfornical organ (SFO), a sensory circumventricular organ [90]. These findings indicated that SFO oxytocin-expressing cells were represented by microglia. Intriguingly, this novel microglial subtype seems to contribute to a wide range of physiological functions, including reproduction, fluid balance, cardiovascular and neurological regulation.

In response to lipopolysaccharide (LPS) stimulation (inflammatory stimulus) of primary human macrophages, the transcription of *OXT* results in upregulated OXTR protein via the activation of NF-κB. In this context, the co-incubation of LPS-stimulated macrophages with OXT has been able to reduce IL-6 secretion [90]. This suggests that elevated expression of OXTR promotes the anti-inflammatory effects induced by OXT.

Additionally, LPS stimulation of primary murine microglia has been demonstrated to increase OXTR expression, as well as the production of TNF-α, IL-1β, COX-2 and iNOS. However, pretreatment of LPS-stimulated microglia with OXT has been able to significantly decrease the production of these pro-inflammatory mediators [91]. Overall, these results are supported by experiments conducted in vivo. In fact, intranasal OXT administration in adult male mice 1 h prior to intra-peritoneal LPS injection was shown to reduce levels of TNF-α, IL-1β, COX-2 and iNOS expression, as well as Iba1 expression in the prefrontal cortex [92].

Despite the aforementioned roles played by OXT, its role in microglia cells is still poorly understood.

Recent findings have recognized that OXT regulates not only a plethora of physiological functions (including reproduction and CNS functions), but also that it can influence the neuronal pathways implicated in the modulation of reactivity of microglial cells during brain development as well as their neuroprotective effect [93].

Yuan et al. reported that OXT was able to modulate the downstream ERK/MAPK pathway in microglial cells [93]. These modulatory effects of OXT could involve various pathways, molecular effectors of OXTR (e.g., NFκB, eEF2 eukaryotic elongation factor 2) or neurotransmitter receptors on the microglial surface membrane [94].

More interestingly, Takayuki Inoue et al. have demonstrated a potential anti-inflammatory effect of OXT on LPS-stimulated microglia through inhibition of the activation of the eIF-2α–ATF4 pathway. These results indicate the anti-inflammatory role of OXT in activated microglia, which would be a potential pathway for developing novel effective strategies by which to regulate the neuroinflammation associated with microglia [95].

Remarkably, an emerging role of OXT in the protection of fetal brain and gastrointestinal system development at birth has been demonstrated via its potent anti-inflammatory effects. In another setting, OXT was shown to decrease brain inflammation not only in postnatal animals but also in adult animals, thus limiting the oxidative stress produced by microglia cells [91,96,97,98,99].

In fact, Mairesse et al. demonstrated a central anti-inflammatory effect of oxytocin receptor agonist on animal models with perinatal brain injury induced by a gestational low protein diet. This effect was reproduced in vitro using primary microglial cell cultures from rats subjected to a gestational low protein diet. The treatment with the OXTR agonist was able to improve myelination, long-term brain connectivity and behavior. This finding reinforces the belief that the modulation of OXT signaling in the developing brain may be an effective approach to counteracting neuroinflammation-induced brain damage of perinatal origin [100].

Moreover, the OXT system could be studied as a molecule with a neuroprotective role in the immature brain [92].

## 5. Effect of Oxytocin in Brain

It is worth noting that recent studies in animals have shown a pivotal role of oxytocin in the regulation of central inflammatory response and neuroinflammation after brain ischemia [86]. During birth, OXT seems to orchestrate many physiological processes in the mother and fetus to warrant all the useful steps for a successful delivery (e.g., fetal analgesia and lung maturation, which increase mother-infant bonding) [101].

In the early phase of life, the brain has great plasticity and changes its shape, which is of high importance for the maturation of accurate excitatory and inhibitory neuronal circuits. Gamma-aminobutyric acid (GABA) is an important inhibitory neurotransmitter in adults that has an excitatory role in the immature brain, whereas during its first postnatal week it switches to an inhibitory action [102,103]. In Fragile X syndrome and several neurodevelopmental diseases, GABAergic signaling is altered [104,105].

OXT has a key role in the biphasic transition of GABA. During parturition, a great amount of OXT is released [105] that could easily cross the placenta and reach the fetus [106]. Tyzio et al. have shown that maternal OXT is necessary to support GABA switching [102], and inhibition of this pathway can result in a phenotype with autistic characteristics in the offspring [107].

Recently, it was reported that OXT acts as an anti-inflammatory molecule in the brain, where it reduces inflammation, oxidative stress, and pro-inflammatory cascades mediated by microglia [92,96,97]. Similar effects have been reported in the endothelial cells of postnatal and adult animals [90].

Karelina et al. have demonstrated an ischemic stroke in experimental animal models, wherein endogenous hypothalamic OXT is released, the size of infarct is reduced and antioxidant activity is increased after focal cerebral ischemia [97]. Additionally, the authors have shown that pretreatment with an OXTR antagonist blocks these protective effects in socially housed mice [97].

Recent evidence has shown that microglia activation occurs in autistic brains [108,109,110]. More interestingly, in recent studies it has been demonstrated that the administration of OXT improved the behaviors of autistic mice, reducing anxiety, depression and repetitive behavior, and ameliorating social interaction. In autistic mice, oxidative stress and inflammation have been reduced after treatment with OXT. Indeed, treatment with OXT has reduced activated microglia in the hippocampus and amygdale of autistic mice [111].

The prosocial effects of OXT have been observed in genetically and phenotypically diverse mouse models of autism-relevant behaviors, leading to the belief that OXT could have generalized efficacy across subtypes of autism spectrum disorders [112,113]. Moreover, a recent study demonstrated that OXT metabolite, but not synthetic OXT receptor agonists, could induce prosocial effects in an autistic mouse model [114].

Due to its importance in modulating synaptic plasticity, a failure of the OXT system during early development could impact social behavior by altering synaptic plasticity in brain regions implicated in social behavior.

Prader–Willi Syndrome (PWS) is a multisystem disorder that appears during the neurodevelopmental phase and includes abnormal clinical features during development such us acute hypotonia and feeding problems in infants, followed by incessant feelings of hunger leading to obesity [115]. Individuals with PWS also manifest intellectual disabilities [116], with their numbers of PVN-OXT neurons reduced and levels of circulating OXT decreased [117]. The intranasal administration of OXT in individuals under age of six months with PWS has ameliorated feeding and social skills [118], confirming that alterations in the OXT system may be imputable to deficits in the social behavior of PWS, and that OXT treatment may have beneficial effects on this disorder. The efficacy of intranasal OXT remains a topic of debate, considering that these results have not yet been confirmed by other studies [119,120].

Mutations in the contactin-associated protein-like 2 (CNTNAP2) neuronal transmembrane protein that belongs to the neurexin family are associated with different deficits such us cortical dysplasia-focal epilepsy (CFDE), attention-deficit hyperactivity disorder (ADHD) and autism spectrum disorders (ASD) [121,122,123].

Mice with a deletion of the Cntnap2 gene exhibit a significant and specific reduction in the number of cells that express OXT in the PVN, as well as the OXT concentrations in brain extracts. The intraperitoneal or intranasal administration of OXT in these mice ameliorates their social behavioral deficits [124].

The neuroprotective effect of OXT was also demonstrated in a tMCAO rat model, where it was demonstrated that the positive effects of OT on the outcomes of ischemic stroke may be associated with a reduction of calpain –1 expression [125].

In primary rat neural cells exposed to OXT before induction of an experimental acute stroke, it was demonstrated that OXT exerts neuroprotection by altering the expression patterns of the GABA_A_R subunit and the kinetics of GABA-induced chloride ion influx. It was also shown that the introduction of OTX during acute stoke did not induce neuroprotection. OXT could be used as a pharmacological ischemic preconditioning factor that can attract GABA_A_R towards neuroprotection [126]. After facial nerve crush injury in rats, OXT had also a good outcome, demonstrating a good capacity for the neuro reparative process [127].

In developed countries, stress-related disorders such as anxiety and depression are increased. Different studies have found a correlation between OXT concentrations in blood and some stress-related disorders such as post-traumatic stress disorder (PTSD), anxiety disorders and depression [128,129].

In patients with depression, the concentration of OXT has been shown to be lower compared to control subjects [130,131].

It has also been shown in rats and mice that endogenous OXT or exogenous OXT has antidepressant effects [132,133].

Wang et al. found that in a rat model of postpartum depression, the mRNA expression of OXT in the PVN was lower compared to the control group, and that administration of OXT into the PVN reversed their behavior [134].

Patients with depression, symptoms of depression and separation anxiety during pregnancy have been shown to have lower plasma OXT concentrations [135,136].

The intranasal application of OXT has been proven to increase prosocial behaviors in patients with social anxiety disorder or lower levels of anxiety [137]. OXT regulation plays an important role also in metabolic diseases, and anorexia nervosa (AN) is one of these. AN is a metabo-psychiatric disorder with a high mortality rate [138]. Different regulatory proteins are very important in the monitoring of food intake, affecting homeostatic, mainly hypothalamic control of feeding. Emotions, physical activity motivation and reward assessment are determinants for the regulation of food intake and AN etiology [139].

The importance of OXT in metabolism regulation and food intake has been confirmed by the presence of OXT receptors in the pancreas, gastrointestinal tract and adipocytes [140,141]. Additionally, it was demonstrated that OXT-deficient or OXT receptor-deficient animals have an increased food intake and body weight [142]. In these animals, the central or peripheral OXT administration has significantly attenuated the intake and led to sustained animal body weight reduction [143].

Furthermore, other research findings by Peris at al. have demonstrated that pro-opiomelanocortin neurons (POMC) are activated by OXT that acts as the downstream mediator of the leptin effects. According to the authors, OXT might attenuate eating behavior by modulating reward-related signaling [144]. More interestingly, it was demonstrated that OXT administration can lead to a reduction in caloric intake and consumption of palatable meals, as well as improved glucose and lipid metabolism, in both healthy-weight and obese subjects [145,146]. Administration of OXT via the intranasal route can reduce body weight in obese adults, but not in obese children, demonstrating that there is an age-related response to OXT and that younger individuals might respond differently to OXT than older ones [147]. In female patients with AN, an association was found between genetic variations in the OXT receptor and eating disorders. In contrast to preview studies, a recent research illustrated that in the serum of adolescent inpatients with AN, OXT was higher and was not normalized with partial weight recovery. In contrast to preview studies, a recent research illustrated that in the serum of adolescent inpatients with AN, OXT was higher and was not normalized with partial weight recovery; this has led to the hypothesis that OXT could contribute the etiopathogenesis of AN [119,148].

## 6. Strategies to Enhance OTX Delivery

The importance of OXT administration to modulate different stress-related disorder has helped reveal new strategies for enhancing its delivery. Antidepressant drugs and OXT are pharmacodynamically different, but their pathways and molecular mechanisms are shared.

Blood Brain Barrier (BBB) is present in the central nervous system (CNS) and is composed by the endothelial cells of blood capillaries. Unlike capillaries present outside the CNS, blood capillaries of BBB are not fenestrated and contain tight junctions [149]. Moreover, pericytes and astro-glial endfeet that surround endothelial cells.

This particular histological structure is a functional barrier between the interstitial fluid of the brain and the blood; the barrier preserves the controlled biochemical environment that is fundamental for neural function.

Peptides with low molecular weights may enter the CNS through carrier-mediated transport (CMT), which requires an influx and efflux of molecules through membrane solute carrier proteins, a family of proteins involved in carrying neurotransmitters, organic solutes and drugs across the BBB [150]. These membrane transporters help peptides to interact with endothelial cells and alter the permeability of the BBB.

Peptides with high molecular weights and regulatory proteins such as cytokines utilize receptor-mediated transport systems to cross the BBB via receptor-mediated transcytosis (RMT).

Through, RMT, a peptide that normally does not cross the BBB is incorporated with a transportable peptide, and the chimeric complex crosses the BBB via transcytosis.

Peptide drugs are often administered intravenously because of a poor oral bioavailability (e.g., enzymatic degradation and limited absorption by the gastrointestinal system [151,152]).

To improve peptide bioavailability, different processes that modify the surfaces of molecules have been studied.

Liposomes, solid lipid nanoparticles or polymeric nanoparticles [153,154,155,156], are also used to encapsulate neuropeptides and carry molecules to cross the BBB. Particular focus has been given to the use of metal nanoparticles to improve the delivery of peptides to the CNS for treatment of neurological and psychiatric disorders.

Systemically administered OXT arrives at the cerebrospinal fluid (CSF), but with poor BBB penetrance (i.e., on the order of 0.001% CSF recovery of the delivered dose [157]).

Intranasal administration of OXT has led to an increased level of OXT in both the hippocampus and the amygdala of rats and mice [158].

When OXT is intranasally administered, it reaches circulation and the CSF, but one of the unanswered questions was whether it can reach its effective concentration in the brain to produce its behavioral effects.

Yamamoto et al. have shown that after intranasal, intravenous or subcutaneous administrations, OXT levels augmented the CSF in some brain areas, including the amygdala and hypothalamus through a receptor for advanced glycation end-products (RAGE) expressed on brain capillary endothelial cells. It is probable that intranasal administration of OXT is absorbed subsequently into circulation, then transported by RAGE-mediated mechanisms into the CSF [159].

Different studies have shown that intranasal administration of OXT ameliorates depression-like behavior in rats [160] and in human social behaviors [161].

In addition to administration of OXT, many developing technologies have aimed to enhance endogenous brain oxytocin through genetic editing.

Genetic manipulations of hypothalamic magnocellular neurons could be achieved, and may result in cell-specific gene expression of OXT [162], but gene-editing techniques are in the early stages of development that represent an experimental tool to demonstrate the endogenous central OXT system.

## 7. Conclusions

**Figure 1:**
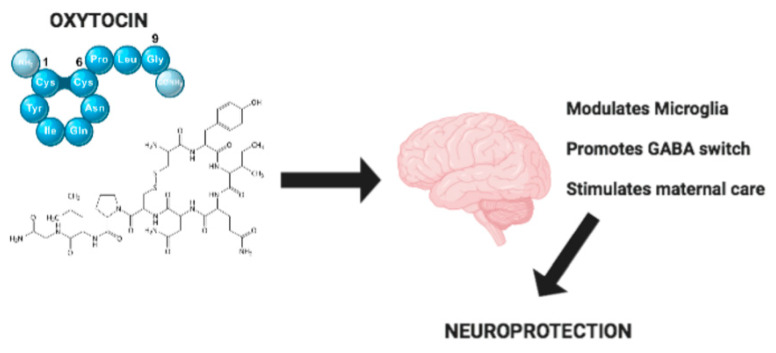
Mechanisms of Oxytocin neuroprotection.

A number of studies have underlined that neuroinflammation associated with abnormal microglia reactivity plays an essential role in the etiology and progression of neurodegenerative diseases. It is important to stress that, in this context, OXT seems to play an important positive role, as reported in Figure 1. The effects of OXT are not only limited to a modulatory action on neuroinflammation, but also include the development of correct neural circuitry. Indeed, OXT regulates the “GABA switch”, which is linked to the onset of neurodevelopmental disorders. Moreover, OXT mediates neuroprotection through a regulation of maternal behavior and alterations of maternal care. OXT also has an important role in modulating synaptic plasticity and stress-related disorders such as depression and anxiety, which are very widespread in developed countries. Also, in metabolic diseases such as anorexia nervosa, OXT regulation is critical. Due to these important functions, strategies to enhance the delivery of OXT must be improved to ensure that OXT can reach its target receptor OTXR in the CNS. Therefore, we conclude that OXT could constitute a potential therapeutic strategy for the treatment of neurodegenerative and neurodevelopmental disorders through its anti-inflammatory and neuroprotective properties.
